# The Development of Cortical Responses to the Integration of Audiovisual Speech in Infancy

**DOI:** 10.1007/s10548-023-00959-8

**Published:** 2023-05-12

**Authors:** Aleksandra A. W. Dopierała, David López Pérez, Evelyne Mercure, Agnieszka Pluta, Anna Malinowska-Korczak, Samuel Evans, Tomasz Wolak, Przemysław Tomalski

**Affiliations:** 1 Faculty of Psychology, University of Warsaw, Warsaw, Poland; 2 Department of Psychology, University of British Columbia, Vancouver, Canada; 3 Institute of Psychology, Polish Academy of Sciences, Warsaw, Poland; 4 Goldsmiths, University of London, London, UK; 5 Kings College London, London, UK; 6 University of Westminister, London, UK; 7 Institute of Physiology and Pathology of Hearing, Bioimaging Research Center, World Hearing Centre, Warsaw, Poland

**Keywords:** fNIRS, Audiovisual integration, Infant, Speech processing, Neurocognitive development

## Abstract

In adults, the integration of audiovisual speech elicits specific higher (super-additive) or lower (sub-additive) cortical responses when compared to the responses to unisensory stimuli. Although there is evidence that the fronto-temporal network is active during perception of audiovisual speech in infancy, the development of fronto-temporal responses to audiovisual integration remains unknown. In the current study, 5-month-olds and 10-month-olds watched bimodal (audiovisual) and alternating unimodal (auditory + visual) syllables. In this context we use alternating unimodal to denote alternating auditory and visual syllables that are perceived as separate syllables by adults. Using fNIRS we measured responses over large cortical areas including the inferior frontal and superior temporal regions. We identified channels showing different responses to bimodal than alternating unimodal condition and used multivariate pattern analysis (MVPA) to decode patterns of cortical responses to bimodal (audiovisual) and alternating unimodal (auditory + visual) speech. Results showed that in both age groups integration elicits cortical responses consistent with both super- and sub-additive responses in the fronto-temporal cortex. The univariate analyses revealed that between 5 and 10 months spatial distribution of these responses becomes increasingly focal. MVPA correctly classified responses at 5 months, with key input from channels located in the inferior frontal and superior temporal channels of the right hemisphere. However, MVPA classification was not successful at 10 months, suggesting a potential cortical re-organisation of audiovisual speech perception at this age. These results show the complex and non-gradual development of the cortical responses to integration of congruent audiovisual speech in infancy.

**Supplementary Information:**

The online version contains supplementary material available at 10.1007/s10548-023-00959-8.

## Introduction

From birth, we experience and learn about speech in a multisensory context. After birth, newborns can distinguish between congruent and incongruent (mismatched auditory and visual) audiovisual speech (Aldridge et al. 1999), while five-month-olds can integrate audiovisual syllables (e.g., Rosenblum et al. 1997). With increasing age, the mechanisms supporting integration of audiovisual speech change. Younger infants rely on low-level cues (e.g., temporal synchrony) to integrate audiovisual speech while older infants rely on higher-level cues (e.g., gender, Patterson and Werker 2002) (Lewkowicz and Ghazanfar 2009).

The developmental changes in neural correlates of integration of audiovisual speech remain understudied. While multiple studies focused on a single age group, to the best of our knowledge, only a single study looked at the development of neural responses to integration across a broader period in the second half of the first year of life. Kushnerenko and colleagues (2013) measured visual attention and event-related potentials to congruent and incongruent syllables in a group of 6- to 9-month-olds (Kushnerenko et al. 2013, see also Hyde et al. 2011; Reynolds et al. 2014; Riva et al. 2022). Infants who show less developmentally mature looking patterns to audiovisual speech (i.e., looking mostly at eyes) showed an audiovisual mismatch response (AVMMR, Kushnerenko et al. 2008). On the other hand, infants who showed a more developmentally mature looking pattern (i.e., looking mostly at the mouth) did not show the AVMMR (just like adults). The neural correlates of speech processing change across infancy, with some studies showing increasingly left-lateralised responses to auditory speech (e.g., Minagawa-Kawai et al. 2007). The frontal and temporal regions are active during perception of audiovisual speech (Altvater-Mackensen and Grossmann 2018; Egorova et al. 2010; Fava et al. 2014; Lloyd-Fox et al. 2015; Mercure et al., 2020) and integration of incongruent audiovisual speech (Altvater-Mackensen and Grossmann 2016; Ujiie et al. 2020). At 5 months of age, the left inferior frontal region was more active to congruent than incongruent vowels (Altvater-Mackensen and Grossmann 2016). Around 9 months of age, congruent audiovisual syllables elicited left temporal, while incongruent elicited bilateral temporal activations (Ujiie et al. 2020). Together, these studies show that the fronto-temporal network supporting integration of audiovisual speech is functionally active already in infancy and changes within the first year of life. But, the nature of the cortical responses to integration and their developmental transitions are unclear.

In adults, integration of audiovisual speech elicits non-linear responses (Callan et al. 2003; Calvert et al. 2000; Erickson et al. 2014; Matchin et al. 2014). In particular, activation to multisensory stimuli is higher (super-additive response) or lower (sub-additive response) than the combined activation to each unimodal stimuli (Stein and Meredith 1993). This reflects the additional processes involved in integration (Meredith and Stein 1983; Stein and Meredith 1993). It is likely that during infancy, with increasing behavioural specialisation for processing and integration of audiovisual speech, there are changes in the functional cortical organisation that supports these abilities. As a result of these changes, the non-linear responses to integration likely emerge. However, previous research offers a limited understanding of how the network for integration of audiovisual speech develops. The role of inferior frontal and superior temporal cortex in processing audiovisual speech between 6 months and 9 months of age – a time of important phonological development (e.g., Werker and Tees 1984) – has never been tested. Studies that measured integration of audiovisual speech in infants focused on neural responses to incongruent audiovisual speech (Altvater-Mackensen and Grossmann 2016; Kushnerenko et al. 2008, 2013; Ujiie et al. 2020), rather than the non-linear (super- and sub-additive) responses to integration of congruent audiovisual speech.

In this pre-registered study (Dopierała et al. 2020), we investigated the development of fronto-temporal responses to integration of audiovisual speech between 5 and 10 months of age. We selected these age points to reflect the neural responses right before the onset of specialisation for native speech (around 5 to 6 months) and right after the specialisation (9 to 10 months) (e.g., Werker and Tees 1984). We compared fronto-temporal responses to the presentation of audiovisual speech with the responses to the presentation of consecutive auditory and visual speech. To shorten testing time and decrease attrition rates, we combined auditory and visual speech within a single condition - the alternating unimodal condition (Olson et al. 2002). There was a 600 ms lag between the onset of the auditory syllable and visual articulation in that the audible speech sound never overlapped with the visible mouth movement. Adults asked about their experience indicated that they perceived the alternating syllables as separate instances of speech. We proposed that differential activation to the bimodal relative to alternating unimodal condition would reflect integration. Our pre-registered hypotheses (Dopierała et al. 2020) predicted that the superior temporal region would show a different response to the bimodal than alternating unimodal conditions, however these responses would only emerge around 10 months of age. In that, at ten, but not at five months of age (1) specific channels would show different responses to bimodal (audiovisual) relative to alternating unimodal (auditory + visual) speech, and (2) widespread patterns of cortical responses would be successfully classified based on distributed patterns of activation (see below) as either to bimodal (audiovisual) or alternating unimodal (auditory + visual) speech. (Additional pre-registered hypotheses (Dopierała et al. 2020) are reported in the Supplementary Material.)

In infancy cortical regions respond preferentially to particular stimuli categories (e.g., Altvater-Mackensen and Grossmann 2016). However, infants’ cortical responses are less stable or marked than adults’ (Deen et al., 2017) and therefore may be missed by standard channel-by-channel analyses (Emberson et al. 2017). One way to address this problem is using Multivariate Pattern Analysis (MVPA). MVPA harnesses weakly discriminative information that is distributed over multiple channels and can therefore, in some cases, provide greater sensitivity than the univariate general linear model (Haynes and Rees 2006). MVPA considers the spatial pattern of activation across multiple channels allowing us to ask whether information about specific stimuli can be extracted from these multi-channel patterns. This allows us to investigate how activity across multiple brain regions contributes to a cognitive process. Therefore, to gain a better understanding of the development of cortical responses to integration of audiovisual speech, we used both standard univariate analyses - repeated-measures ANOVA (RM-ANOVA, e.g., Grossmann et al. 2010) - and novel multivariate analyses, only recently adapted for use with developmental fNIRS data (Emberson et al. 2017; Mercure et al., 2020).

## Methods

### Participants

The final sample included 42 infants: 20 in the younger age group and 22 in the older age group (see Table 1 for detailed sample characteristics). Although the initially planned sample size of 46 infants (Dopierała et al. 2020) could not be achieved due to the onset of the COVID-19 pandemic and related lab closures, the final sample had sufficient power (0.85 and 0.89 in younger and older age groups respectively) to detect medium-sized effects (f = 0.25, RM ANOVA, within factors) (G*Power software, Faul et al. 2009)^1^. All infants in the final sample were born full term, they were typically developing with no vision or hearing deficits, monolingual (or had less than 30% of daily exposure to another language), and came from Polish-speaking families. An additional 34 infants were tested but excluded due to age outside the age range (N = 3), technical difficulties (N = 3), improper headgear fitting (N = 7), experiment suspended due to infant behaviour (fussiness, crying, excessive movement, N = 6), not looking at the screen for at least 60% of the required minimum number of trials (3 per experimental condition, N = 3), infant taking off/moving headgear and/or pulling out fibres during testing (N = 4), or failure to reach trial and channel inclusion criteria (N = 2, N = 6, see Sect. 2.4). The study was approved by the Research Ethics Committee at the Faculty of Psychology, University of Warsaw, Poland, and conformed with the standards of the Declaration of Helsinki. Prior to the testing session, parents gave written informed consent. For their participation, the families received a diploma, a small gift (a baby book), and a video recording of their play in the laboratory.

**Table 1:** Final sample characteristics

Age group	Younger age group	Older age group
Number, female	20, 8 F	22, 8 F
Age, months	5.8 (0.49) [5.2–6.7]	9.7 (0.56) [9.0–10.7]
Head circumference, cm	44.46 (1.53) [42–48]	45.84 (1.35) [43–48]†
Headband size: small/large	19/1	16/6†
Gestational age, weeks	39 (1.47) [36–41]	39 (1.26) [36–41]
Channels included	45 (1.57) [41–16]	43 (3.6) [32–46]
Number tested during COVID-19	3	7

*Note*. Mean (SD) [range]. All infants were monolingual (or had less than 30% of daily exposure to another language). †Due to sanitary procedures head measurements of infants tested during the pandemic were not taken and they all wore a small headgear

### Stimuli

**Fig. 1:**
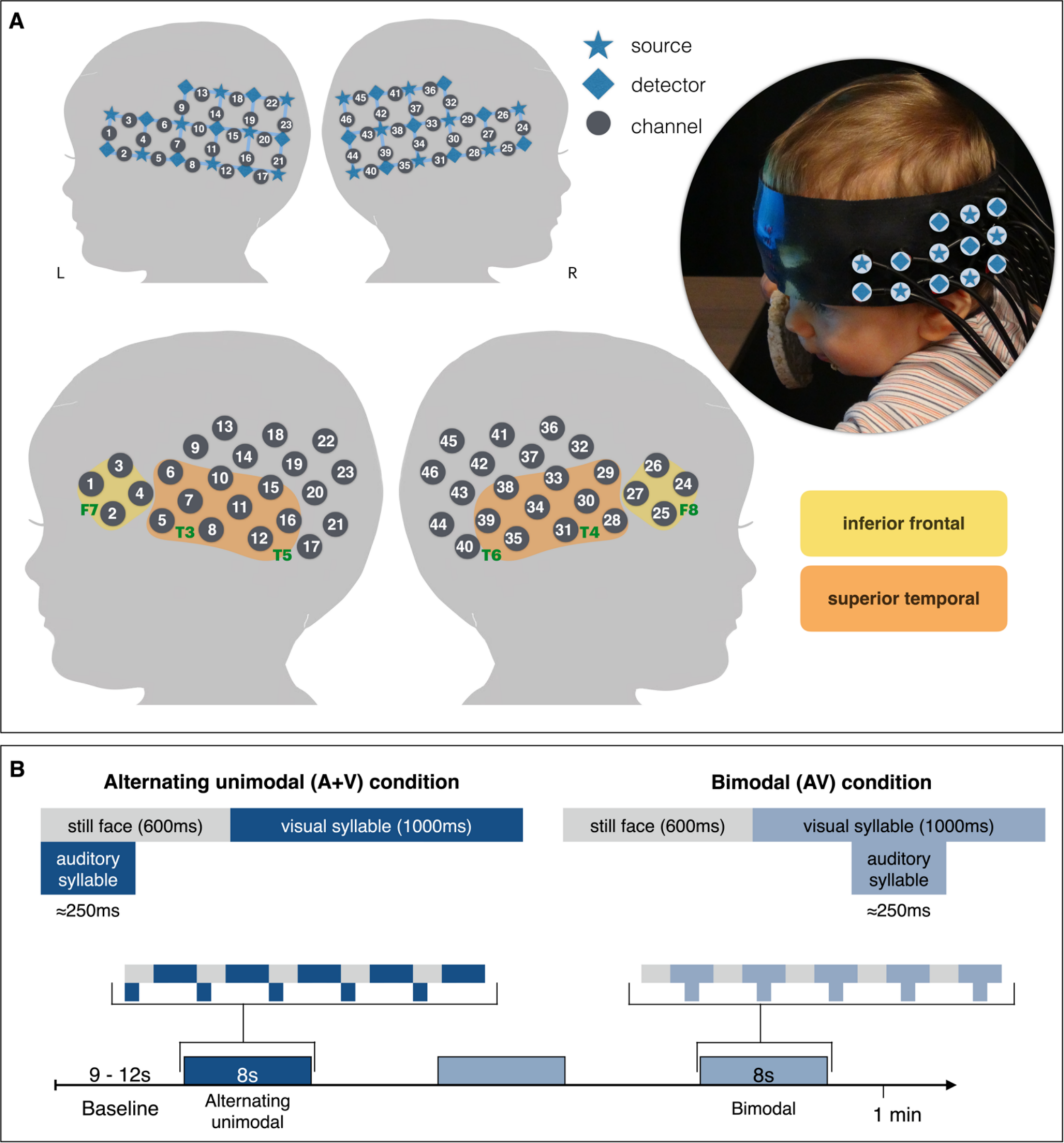
FNIRS headgear and experimental paradigm. (**a**) Picture of an infant wearing the NTS fNIRS headgear and illustrations of channel location in relation to infant’s head: highlighted sources (stars) and detectors (diamonds), grey circles indicate measurement channels and channel numbers with the 10–20 coordinates superimposed on the diagram in green. Channels within the yellow box are part of the inferior frontal region, orange - superior temporal region. (**b**) Experimental stimuli design and study paradigm

Stimuli were created from two video clips of female native polish speakers looking directly at the camera (i.e., eye gaze fixed at the infant), shown from the neck up against a dark grey background. The only part of the face changing was the visible articulation of syllables /ba/ and /ga/. Stimuli were edited with Davinci Resolve, version 15 software (BlackmagicDesign, Australia) to create two experimental conditions: alternating unimodal (auditory + visual) and bimodal (audiovisual) speech. The alternating unimodal (auditory + visual) speech condition started with the auditory syllable presented simultaneously with the still frame of the speaker’s face (first frame of the clip), followed by silent visual syllable, see Fig. 1B. The visible articulation started 600ms after the onset of the auditory syllable (~ 250ms), thus there was no temporal overlap between the audible speech sound and the visible articulation. The bimodal (audiovisual) speech condition started with a silent still frame of the speaker’s face, followed by concurrent audiovisual syllable (/ba/ or /ga/) see Fig. 1B. Stimuli were presented in 8s-long trials including 5 repetitions of a single stimuli, alternating with a silent dynamic baseline, see Fig. 1B. To create baseline stimuli, we used a still frame from the blurred, pixelated, and muted video clips (edited with Movavi Video Editor software, version 15, Movavi, USA). To create a perception of motion the still frames were edited to slowly zoom in, creating 3s long videos. Baseline trials had jittered length: 9–12s (e.g., Lloyd-Fox et al. 2011). Trials were presented in a pseudo-random order, 3 trials per experimental condition every two minutes (e.g., Lloyd-Fox et al. 2011).

### Procedure

Infants sat on their parent’s lap, approx. 60 cm from a screen, in a dimly lit room. We gently placed the fNIRS headgear on the infant’s head, aligning the midline to the infant’s nasion, and placing the sides so that the midpoint of the lower row of channels was above the pre-auricular points (Lloyd-Fox et al. 2009), see Fig. 1A. Infants wore different headgear sizes depending on head circumference (see Table 1). We instructed parents to refrain from talking to or interacting with the baby throughout the procedure. To draw the infant’s attention to the screen and away from the headgear being placed on their head, the experiment started with a movie of an aquarium. Infants wore a custom build CBCD fNIRS headgear (http://cbcd.bbk.ac.uk/node/165), consisting of two source-detector arrays (Fig. 1A) with 46 channels (source-detector separations; 2 cm) covering the frontal, fronto-temporal, temporal, temporo-fronto-parietal, and temporoparietal regions (Lloyd-Fox et al. 2014). Once the headgear was in place and the infant was looking at the screen, the experimental task started. We recorded fNIRS data using an NTS optical tomography system (Gowerlabs Ltd. L, UK) with two continuous wavelengths of source light: 780 and 850 nm. On the screen, time-locked stimuli were presented using Psychtoolbox (Brainard 1997; Kleiner et al. 2007; Pelli 1997) for MATLAB version 9.2 (R2017a, Mathworks Inc., Sherborn, MA, USA). The experimenter stood behind a curtain or in an adjoining room, hidden from the infant. Infant behaviour was monitored throughout the procedure, and video-recorded for off-line coding of looking behaviour.

### fNIRS Analyses

Our fNIRS analysis plan was pre-registered (Dopierała et al. 2020). We pre-processed raw fNIRS data in HOMER2 (Huppert et al. 2009) following previously established pipelines and guidelines (e.g., Di Lorenzo, Pirazzoli et al., 2019; Lloyd-Fox et al. 2015). We excluded channels with raw intensities below 0.001µM or above 10µM. Infants who contributed data from less than 31 (of 46) channels were excluded from further analyses (Mercure et al., 2020). To recover most motion-affected trials (e.g., Di Lorenzo, Pirazzoli et al., 2019) we corrected motion artefacts using wavelet analyses (iqr = 0.8) and spline correction (Scholkmann et al. 2010; Molavi and Dumont 2012). As some motion artefacts remained, we excluded channels with excessive motion artefacts (observed on over 3 trials throughout the testing session) and trials containing or preceded by (5s) significant motion artefacts. Additionally, we manually coded infants’ looking behaviour during the experiment and excluded trials during which the infant looked away from the screen for over 60% of the time or when parent interfered (e.g., talked to the baby). Finally, we excluded infants who contributed less than 3 trials per condition (e.g., Mercure et al., 2020). For infants that contributed data to the final sample, we removed physiological noise using a bandpass filer (lpf 0.50 Hz, hpf 0.03 Hz). Then, we converted data to relative concentrations of HbO and HbR, assuming a differential pathway factor of 5.1 (Duncan et al. 1996; Lloyd-Fox et al. 2010). Finally, we segmented data into 25s blocks: 5s pre-stimulus baseline and 20s post-stimulus time period (e.g., Lloyd-Fox et al. 2011, 2015; Mercure et al., 2020). For each infant, we calculated the latency of the peak response within the 20s post-stimulus time period across both chromophores and conditions. When averaged across channels, the response peaked around 9s from stimulus onset. In the pre-registration (Dopierała et al. 2020), we planned to analyse the 8s time window around the peak, however, further inspection of the data revealed variability in observed peak latency depending on the channel. To account for that variability we split the activation time window into two: 5-10s and 10-15s (e.g., Lloyd-Fox et al. 2017). For each infant, condition and channel, we calculated the mean changes in concentration of HbO and HbR (e.g., Gervain et al. 2008) in three time windows: -5–0s pre-stimulus baseline, 5–10s and 10–15s post-stimulus.

### Statistical Analyses

**Fig. 2:**
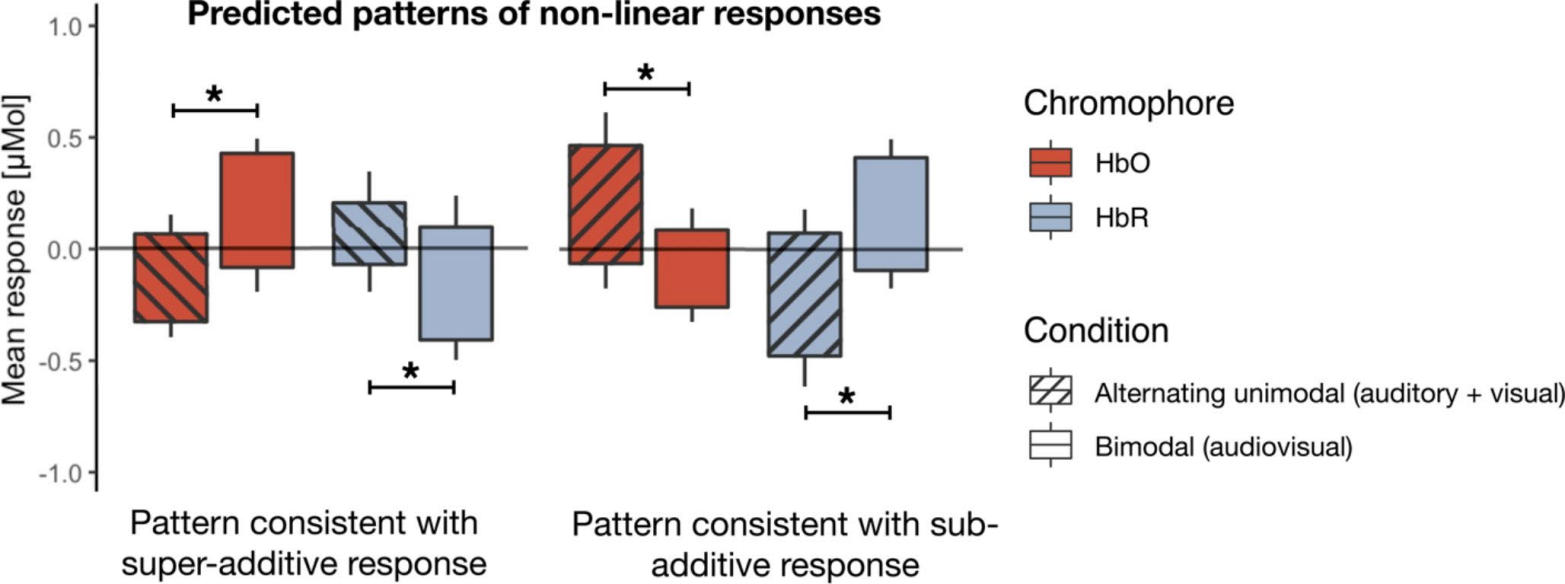
Theoretical pattern of predicted non-linear responses to the alternating unimodal (striped) and bimodal (plain) conditions. If the difference between the conditions was significant, responses were interpreted as either super- or sub-additive. Note that interpretation of the response differed depending on the chromophore: HbO or HbR

We conducted two types of analyses: univariate and multivariate pattern analyses (MVPAs) (Dopierała et al. 2020). For univariate analyses, we used channel-by-channel RM ANOVAs with within factors of time (-5–0s, 5–10s, 10–15s), condition (bimodal (audiovisual) speech, alternating unimodal (auditory + visual) speech), and between factor of age (younger − 5- to 6-month-olds, older − 9- to 10-month-olds). To identify channels showing different response to bimodal than alternating unimodal condition, we used simple planned contrasts: We compared the change between baseline and activation time windows (-5–0s vs. 5–10s, -5–0s vs. 10–15s) between the two experimental conditions (bimodal (audiovisual) speech vs. alternating unimodal (auditory + visual) speech). With planned post-hoc comparisons, we analysed the direction of the difference between conditions (higher to bimodal or alternating unimodal) within each of the two activation time windows. We applied the FDR (Benjamini and Hochberg 1995) correction for multiple comparisons and reported both corrected and uncorrected results. We ran separate analyses for each chromophore: oxygenated (HbO) and deoxygenated (HbR) haemoglobin (e.g., Grossmann et al. 2010). Both an increase in the concentration of HbO and a decrease in the concentration of HbR relative to the baseline period were interpreted as cortical activation in infants (e.g., Lloyd-Fox et al. 2010). We predicted that a greater activation to bimodal than alternating unimodal condition would reflect a cortical response consistent with a super-additive response, while a greater activation to alternating unimodal than bimodal condition reflects a cortical response consistent with a sub-additive response. The interpretation of the response depends on the chromophore: higher HbO increase to bimodal condition reflects a response consistent with a super-additive response, higher HbR increase to bimodal condition reflects a response consistent with a sub-additive response, on the other hand, higher HbO increase to alternating unimodal condition reflects a response consistent with a sub-additive response, higher HbR increase to alternating unimodal condition reflects a response consistent with a super-additive response, see Fig. 2.

For the MVPAs, we used a Support Vector Machine (SVM) to train a classification boundary that separates neural patterns associated with two labeled experimental conditions. Once trained, the model can be tested by assessing its ability to successfully discriminate the conditions, for unseen data, in which the labels of the two conditions have been withheld. If the model is able to successfully predict the labels of the unseen data at a level greater than chance, we assume that the neural representations associated with each stimulus type are distinctly encoded in the neural responses (Haxby et al. 2001; Haxby and Gobbini 2012). The data were z-scored within each channel across all infants to ensure that the channels were in comparable scales for classification. We used a leave-one participant-out approach. The classifier was trained on a balanced training set of neural patterns from all of the participants excluding the to-be-classified participant. The model was trained on the averaged pattern derived from the fNIRS epoch data for each of the two experimental conditions from all participants except one. We used all channels, not just the ones over the inferior frontal and superior temporal regions, as MVPA performs better with greater numbers of channels (Emberson et al. 2017). Not all channels contained usable data for all participants. These channels were dropped out of both the training and test patterns when classifying participants that had missing channels. This meant that the exact channels used in the analysis differed slightly for each participant. This model was used to predict the condition labels of the held-out participant. This was done iteratively for all participants to provide an accuracy estimate (proportion correct) for the set of infants. Then, to establish whether this observed accuracy was higher than chance, we compared whether the classifier accuracy trained on data with true labels was better than the classifier trained on data with randomly shuffled labels (Mercure et al., 2020). To do so, the classifier was trained 1000 times, randomly either maintaining or changing condition labels in the data, shuffling data labels differently at each permutation. The probability value was established by pooling then ranking the observed and permuted accuracies in order to identify the number of times the observed classification accuracy was greater than or equal to the accuracies derived from the shuffled data (Pereira et al. 2009). The observed value was included in both the numerator and denominator for calculating the p value, such that if the classification accuracy observed from the data was higher than all the observed permutation values, this would result in a value of p = 1/1001 (Ruxton and Neuhäuser 2013). For successful classifications, we report which channels contributed most to the classification (the classifier weight value for the channels). The weights for each channel were determined by re-training the classifier using the data averaged over all participants for each condition and extracting the weight vector of a model trained on these averaged patterns. To account for the fact that some channels were dropped out of the classification when calculating the classifier accuracy, due to missing channels, we trained this final model only using channels for which there was usable data in at least 80% of participants. We multiplied the weight vector by the average patterns and visualised the most informative channels, defined as the channels contributing the highest 30% of values. This approach takes into account the channel values, their associated weight and how combining these values influences the classification outcome. The most informative channels were defined as the channels contributing the 30% most extreme values. Note that due to the normalisation, the channels contributing most to classifying in favour of the positive class (e.g. one of the conditions) were the same as those contributing most to classifying the negative class (e.g. the other condition). As such, the weights reflected the channels that provided the most effective discrimination between conditions rather than necessarily characterising one condition or the other. As in univariate analyses, we conducted separate MVPAs on mean changes (across all trials) in HbO and HbR during each activation time window (5–10s and 10–15s post-stimulus onset) for each participant. As we hypothesised that classification would be successful only in older infants, we conducted separate analyses for each age group. To test for hemispheric contributions to classification, we conducted MVPAs separately on all, left, and right hemisphere channels. Multivariate analyses were conducted using a custom Matlab script (https://github.com/speechAndBrains/fNIRS_tools).

Given recent findings pointing to possible differences in language and face processing between infants tested before and during the COVID-19 pandemic (e.g., Huang et al. 2021; for a discussion see Carnevali et al. 2022), we ran additional exploratory analyses. As previous infant fNIRS studies using MVPA found significantly successful classifications in sample sizes below 20 (Emberson et al. 2017 N = 18; Mercure et al., 2020 N = 19), we re-ran the MVPAs on data from infants tested before the pandemic. Unfortunately, the number of infants with usable data (see Sect. 2.1 and 2.4) tested before the pandemic (younger N = 17, older N = 15) was too small for univariate analyses. Post-hoc power analyses assuming medium effect size (f = 0.25, RM ANOVA within factors) found that the achieved power was 0.78 and 0.71 respectively (G*Power software, Faul et al. 2009). Therefore, we could not analyse the pre-pandemic groups using univariate analyses.

## Results

On average, infants in the younger age group contributed 4.35 (SD = 1.04) alternating unimodal (auditory + visual) speech and 4.85 (SD = 0.81) bimodal (audiovisual) speech trials to final analyses; they contributed significantly more bimodal than alternating unimodal trials, z = -2.18, p = .029. Infants in the older age group contributed 5.59 (SD = 1.56) alternating unimodal (auditory + visual) speech and 5.5 (SD = 0.96) bimodal (audiovisual) speech trials to final analyses; the number of included trials was not significantly different depending on the condition, p > .1.

### Bimodal (Audiovisual) Speech Compared to Alternating Unimodal (Auditory + Visual) Speech

To investigate which channels show differential responses depending on the stimuli, we compared responses to bimodal and alternating unimodal speech in each age group (see Sect. 2.5). In the following sections, we focus on the channels in the inferior frontal and superior temporal regions (see Fig. 1) that showed significant effect, for results from all channels see Table 2. For each channel we analysed two time windows 5-10s and 10-15s, for details on the time window where the effect was observed see Table 2. We report first the simple planned contrasts followed by planned post-hoc comparisons, first in the younger and then older age group.

**Table 2:** Channels showing differential response to synchronous audiovisual and asynchronous auditory/visual speech in both age groups. Planned simple contrasts and post-hoc pairwise comparisons

					time x condition					post-hoc		
Chrom	H	ROI	CH	TW	*F*	*df*	*df*_*(Error)*_	*p*	η^*2*^_*p*_	*p*	A vs. S	interpretation
Younger age group												
HbO	L	ST	5	10–15s	5.449	1	16	0.037	0.244	0.034	S > A	super
	L	-	22	10–15s	5.598	1	16	0.031	0.259	0.032	A > S	sub
	R	IF	24*	10–15s	19.692	1	19	< 0.001	0.509	< 0.001	A > S	sub
	R	IF	26*	10–15s	15.102	1	17	0.001	0.47	0.001	A > S	sub
	R	ST	27	5–10s	6.458	1	17	0.021	0.275	0.02	A > S	sub
	R	ST	29	10–15s	5.432	1	15	0.034	0.266	0.037	A > S	sub
	R	-	42	10–15s	8.498	1	19	0.009	0.309	0.008	A > S	sub
	R	-	44	10–15s	6.132	1	19	0.023	0.244	0.024	A > S	sub
HbR	L	IF	3	10–15s	7.226	1	17	0.016	0.298	0.014	S > A	sub
	L	IF	4	10–15s	4.769	1	17	0.043	0.219	0.042	S > A	sub
	R	IF	24	5–10s	4.881	1	19	0.04	0.204	0.038	A > S	super
				10–15s	4.593	1	19	0.045	0.195	0.043	A > S	super
	R	ST	28	10–15s	6.54	1	16	0.021	0.29	0.02	S > A	sub
	R	ST	31	10–15s	4.696	1	15	0.047	0.238	0.048	S > A	sub
	R	-	42	10–15s	5.102	1	19	0.036	0.212	0.038	A > S	super
Older age group												
HbO	R	ST	29	10–15s	4.534	1	16	0.049	0.221	0.042	A > S	sub
	R	-	37	10–15s	4.795	1	18	0.042	0.21	0.04	S > A	super
HbR	L	ST	15	5–10s	6.087	1	18	0.024	0.253	0.026	S > A	sub
				10–15s	7.384	1	18	0.014	0.291	0.015	S > A	sub
	L	-	20	5–10s	8.76	1	20	0.008	0.305	0.008	S > A	sub
				10–15s	6.219	1	20	0.022	0.237	0.022	S > A	sub

*Note.* Chrom, chromophore, H, hemisphere, CH, channel number, TW, time window, A, asynchronous auditory/visual speech, S, synchronous audiovisual speech, A vs. S, direction of difference in mean HbO/HbR concentration between conditions, super, super−additive response, sub, sub−additive response. * Result significant after FDR correction for multiple comparisons

**Fig. 3:**
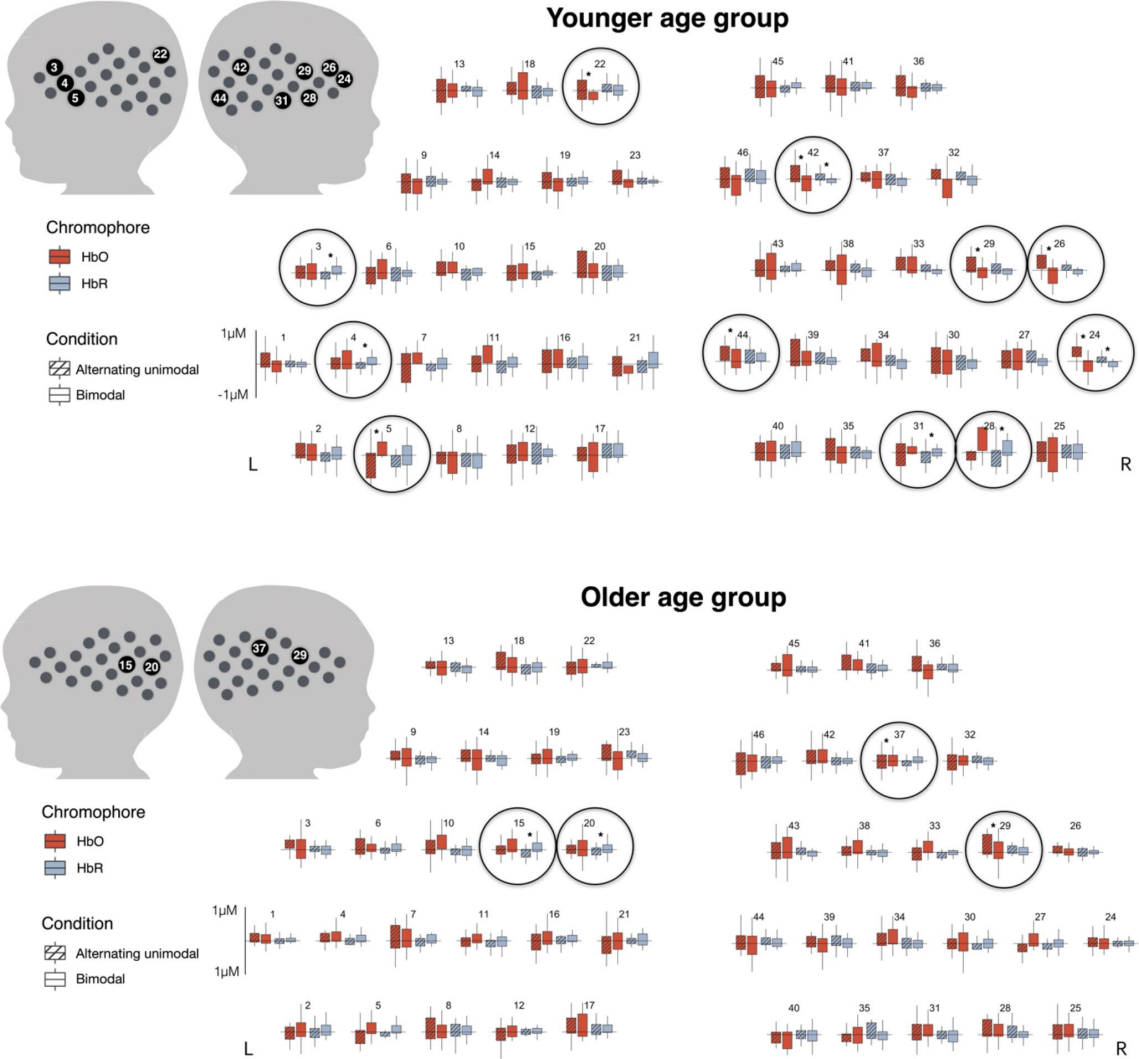
Mean concentration of chromophores (µM, red - HbO, blue - HbR) in the second time window (10-15s) depending on condition: striped - alternating unimodal (auditory + visual) speech, plain - bimodal (audiovisual) speech. Top panel - younger age group, bottom panel - older age group. * p <. 05

*Younger age group.* In the younger age group, the planned simple contrast analyses using F-test (see Sect. 2.5) revealed eight channels showing significantly different responses depending on condition. Two right inferior frontal channels (24, 26) showed significantly different response to bimodal than alternating unimodal speech, which survived the FDR (Benjamini and Hochberg 1995) correction for multiple comparisons p < .05. Furthermore, left inferior frontal (3, 4) and bilateral superior temporal (5, 27, 28, 29, 31) channels showed significantly different responses to bimodal than alternating unimodal speech at an uncorrected threshold p < .05. Planned post-hoc comparisons revealed which of these channels showed super- and sub-additive-consistent responses, see Table 1 and the upper panel of Fig. 3 (note that Fig. 3 shows only responses within the second time window, 10-15s).

One channel showed a response consistent with a super-additive response: Left superior temporal channel (5) showed greater HbO increase to bimodal than alternating unimodal condition.

Nine channels showed responses consistent with sub-additive responses, reflected by either a significant difference in HbO or HbR response. HbO: The right inferior frontal (26) and superior temporal (27, 29) channels, bilateral channels located superior to the superior temporal region (22), and a channel located posterior to the superior temporal region (44) showed greater HbO increase to alternating unimodal than bimodal condition. HbR: the left inferior frontal (3, 4) and right superior temporal (28, 31) channels showed greater HbR decrease to bimodal than alternating unimodal condition.

Finally, two channels showed significant effects in both HbO and HbR concentration. The right inferior frontal (24) channel and a channel located superior to the superior temporal region (42) showed greater HbO and HbR increase to alternating unimodal than bimodal condition.

*Older age group.* In the older age group, the planned simple contrast analyses using F-test (see Sect. 2.5) revealed four channels (15, 20, 29, 37) showing significantly different responses depending on condition. The bilateral superior temporal channels (15, 29), a channel located posterior to the left superior temporal region (20), and a channel located superior to the right superior temporal region (37) showed significantly different responses depending on condition (see Table 2), uncorrected p < .05. None of these channels survived the correction for multiple comparisons. Planned post-hoc pairwise comparisons revealed which of these channels showed responses consistent with super- and sub-additive responses, see Table 1; Fig. 3 (note that Fig. 3 shows only responses within the second time window, 10-15s.

One channel showed a response consistent with a super-additive response. The right hemisphere channel located superior to the superior temporal region (37) showed greater HbO increase to bimodal than alternating unimodal condition).

Three channels showed responses consistent with sub-additive responses, reflected by either a significant difference in HbO or HbR. HbO: A right superior temporal channel (29) showed greater HbO increase to alternating unimodal than bimodal condition. HbR: two left hemisphere channels (15, 20) showed greater HbR decrease to alternating unimodal than bimodal condition.

*Summary*. The simple planned contrasts’ analyses followed up with planned post-hoc comparisons revealed cortical responses consistent with both super- and sub-additive responses in both age groups (5- and 10-month-olds). The responses were predominantly sub-additive-consistent, less widespread in the older than in the younger age group.

### MVPA: Classification of Responses to Bimodal (Audiovisual) and Alternating Unimodal (Auditory + Visual) Speech

Using MVPAs, we compared distributed patterns of mean chromophore concentration (HbO, HbR) for the bimodal and alternating unimodal conditions in each age group (Table S1). In the younger age group, patterns of HbO activation to bimodal AV and alternating unimodal A/V speech in the second time window (10–15s) could be classified at a level greater than chance using right hemisphere channels (*proportion. correct = 0.68, p = .044*). The analysis of channel weights revealed that right frontal and temporal channels (24, 26, 27, 29, 36, 38, 42) contributed most to the correct classification. Patterns of HbR activations in the second time window using right hemisphere channels also reached high classification accuracy (65%) but did not reach statistical significance (*p = .07*). Patterns of HbO or HbR activations in either time window using left hemisphere channels could not be classified successfully (*p > .5*). Although classifiers trained on HbO and HbR responses using all channels achieved high classification accuracy (63%), they were not significant (*p > .1*). Surprisingly, in the older age group, neither patterns of HbO nor HbR responses could be classified at a level greater than chance using all, left hemisphere or right hemisphere channels in either time window (*proportion. correct < 0.55, p* > .3, see Table S1).

#### Exploratory MVPAs Without Infants Tested During COVID-19 Pandemic

To explore whether the unsuccessful classification was related to within-group variability related to COVID-19 pandemic, we re-ran MVPA without infants tested during the pandemic (younger N = 17, older N = 15). In both age groups results were consistent with what we found for the whole group. In the younger age group, consistently with whole-group results, the classification of HbO responses over the right hemisphere in the second time window (10-15s) was successful (*proportion. correct = 0.71, p = .034*), but no other classifications were successful (all ps > 0.1). In the older age group, no classifications were successful (proportion correct < 0.63 > 0.09). Results of all performed classifications are presented in Supplementary materials, Table S2.

## Discussion

The current study is the first cross-sectional investigation of the characteristics, spatial organisation, and development of the cortical responses to integration of congruent audiovisual speech in infancy. Non-linear (super- and sub-additive) cortical responses are a hallmark of integration of audiovisual speech and have been well documented in adults (e.g., Calvert et al. 2000; Wright et al. 2003). Documenting non-linear responses over wide areas of the cortex in infants around 5 and 10 months of age, this study contributes significantly to our understanding of the development of brain specialisation for integration of multisensory stimuli. By contrasting cortical responses to audiovisual speech (bimodal condition) with combined response to auditory and visual speech (alternating unimodal condition), we found that integration of audiovisual speech elicits non-linear cortical responses in 5- and 10-month-olds (at an uncorrected level), consistent with both super- and sub-additive responses observed in adults. In both age groups, observed responses were predominantly consistent with sub-additive responses, which may be a developmental response to integration. Univariate analyses showed that the spatial distribution of responses was different in the younger and older age groups. In the younger age group the non-linear responses were widespread, observed over the inferior frontal and superior temporal regions bilaterally, but predominantly in the right hemisphere. In the older age group the non-linear responses were more focal (observed over a few channels), mostly over the superior temporal region bilaterally. Surprisingly, MVPAs revealed that responses to bimodal versus alternating unimodal speech could be successfully classified in the younger age group, but not the older. These findings show that the neural correlates of integration of congruent audiovisual speech change between 5 and 10 months of age, indicating that the development of audiovisual integration of speech is a protracted and complex process that involves a re-organisation during the period of perceptual attunement for the native language. While complex, our findings offer important new insight into the emergence of non-linear responses to integration of speech in an age period when specialisation for speech develops, i.e., 5- to 10 months. In the following sections we first describe the characteristics and development of non-linear responses in infancy followed by discussion of the development of the spatial organisation of brain regions involved in integration.

### Development of non-linear Responses to Integration of Audiovisual Speech

This is the first fNIRS study to show the development of audiovisual integration of speech in infancy. For the first time, we show that non-linear responses, consistent with both super- and sub-additive responses observed in adults, are observed already in infancy. In both age groups, univariate analyses revealed specific channels showing a significantly different response to the bimodal (audiovisual) than alternating unimodal (auditory + visual) condition. This result is consistent with previous studies, which showed differential responses to congruent than incongruent audiovisual speech (Altvater-Mackensen and Grossmann 2016; Ujiie et al. 2020), differential responses to audiovisual than auditory and audiovisual than visual speech (Altvater-Mackensen and Grossmann 2018), and differential responses to bimodal versus unimodal non-social stimuli (Hyde et al. 2010). In both age groups, single channels showed higher responses to bimodal (audiovisual) than alternating unimodal (auditory + visual) speech, i.e., responses consistent with super-additive responses. This result informs previous EEG findings with 3-month-olds that showed likely super-additive-consistent responses to shapes paired with pure tones (Hyde et al. 2010). Here, we extend these findings to social stimuli, and elucidate the development of non-linear responses to integration in a cross-sectional sample of older infants (around 5 and 10 months). Super-additive responses have been previously observed in adults (e.g., Calvert et al. 2000; Wright et al. 2003). The similarity between infant and adult responses implies an early cortical specialization for integration of audiovisual speech.

Interestingly, while in adults integration of congruent audiovisual speech elicits predominantly super-additive responses (e.g., Calvert et al. 2000), in our study most of the observed responses in infants were consistent with sub-additive responses. Interpretation of sub-additive responses is less straightforward than super-additive responses. Following, we propose three possible explanations for this finding drawing from adult and infant studies. Firstly, in adults when audiovisual stimuli are attended they elicit a super-additive response, while when attention is directed to another source of information they elicit sub-additive response (De Meo et al., 2015). If the observed responses consistent with a sub-additive response – i.e., greater activation to alternating unimodal than bimodal condition - were related to attention, we would expect to see greater attention to the alternating unimodal condition. We measured infants’ visual attention to the presented stimuli and excluded trials when infants looked away. We found that, if anything, infants attended more to the bimodal than alternating unimodal condition: In the younger age group, infants contributed more trials to the bimodal than alternating unimodal condition, while in the older age group the number of trials was not significantly different between the conditions. Thus, it is unlikely that the observed responses consistent with sub-additive responses were related to differences in attention to the two experimental conditions.

Secondly, it is possible that the sub-additive-consistent responses were triggered by perception of the temporal asynchrony between the visual and the auditory syllables or the incongruence between the still face and the auditory syllable (or silence and visual syllable) in the alternating unimodal condition. In adults, sub-additive responses have been mostly observed to incongruent or temporally offset audiovisual stimuli (Calvert et al. 2000; Meredith and Stein 1983; Wright et al. 2003). However, we find this explanation unlikely for three main reasons: (1) we did observe super-additive-consistent responses over some channels in both age groups; (2) the lag between auditory and visual speech (600 ms) was long enough to prevent perceptual integration (Lewkowicz 2010); and (3) observed sub-additive-consistent responses are consistent with previously observed responses to integration of audiovisual speech. At 5 months of age incongruent audiovisual speech elicited smaller inferior frontal response than congruent audiovisual speech (Altvater-Mackensen and Grossmann 2016). Therefore, we assume that infants processed the alternating unimodal condition as auditory and visual speech. Thirdly, the predominance of sub-additive responses may be a developmental pattern. In that, initially integration of speech elicits both super- and sub-additive responses, while the predominance of super-additive responses emerges only after 10 months of age. We would like to see this finding investigated further to see whether the sub-additive responses are consistently observed during integration of congruent speech in infants and whether the predominance of super-additive responses emerges later in infancy.

We compared responses to congruent bimodal audiovisual speech with alternating unimodal speech and found differential responses over bilateral, but predominantly right, fronto-temporal regions at 5 months of age and bilateral superior temporal regions at 10 months of age. By comparing congruent audiovisual speech with alternating unimodal speech, we were able to measure the cortical response to the exact same stimuli rather than a novel, unknown percept (an incongruent stimulus). That is, both the bimodal and alternating unimodal conditions included exactly the same stimuli in either modality. The only difference between the conditions was the temporal overlap, in the alternating unimodal condition there was no overlap between the auditory and visual stimuli. As in adults integration of congruent and incongruent speech elicits different responses (Erickson et al. 2014), our findings showing neural correlates of integration of congruent speech inform the body of research on integration of incongruent speech in infancy (e.g., Kushnerenko et al. 2008, 2013). In line with previous studies showing an audiovisual mismatch response (i.e., differential response to congruent and incongruent syllable) in both 5- and 12-month-olds (Kushnerenko et al. 2008, 2013; Riva et al. 2022) by showing the non-linear response to integration of congruent speech in both 5- and 10-month-olds. Note that while technically asynchronous (visible articulated started 600ms after the onset of auditory speech sound), we employed a much larger time lag than previous studies that measured response to detection of asynchrony. Our findings further inform previous studies on processing asynchrony and incongruency between auditory and visual stimuli (Hyde et al. 2011; Kopp 2014; Kopp and Dietrich 2013; Reynolds et al. 2014). Furthermore, the observed differential responses to bimodal versus alternating unimodal speech are in line with differential ERPs to incongruent and asynchronous speech versus congruent and synchronous audiovisual speech (Hyde et al. 2011; Reynolds et al. 2014), as well as asynchronous versus synchronous non-speech social stimuli (video of a person clapping, Kopp 2014; Kopp and Dietrich 2013) in infants around 5–6 months of age. Moreover, by using fNIRS - which has higher spatial resolution than EEG - we were able to extend these findings to elucidate the spatial organisation (see Sect. 4.2) of the cortical network for speech integration, a goal often cited as an important direction for further research (e.g., Hyde et al. 2016).

### Organisation of the Cortical Network for Integrating Audiovisual Speech in Infancy

Our results further revealed that the non-linear responses to congruent audiovisual speech are observed in the inferior frontal and superior temporal regions in 5- and 10-month-olds. These results are consistent with previous studies which showed bilateral inferior frontal and superior temporal sensitivity to auditory and audiovisual speech (around 5 months of age: Altvater-Mackensen and Grossmann 2016; Cristia et al. 2014; Egorova et al. 2010; Lloyd-Fox et al. 2015; Mercure et al., 2020; Naoi et al. 2012; around 10 months of age: Homae et al. 2007; Naoi et al. 2012; Ujiie et al. 2020; in adults e.g., Callan et al. 2003; Calvert et al. 2000; Erickson et al. 2014; Matchin et al. 2014) and non-speech audiovisual integration already in infancy (Werchan et al. 2018). As we tested two age groups, our results extend previous studies by showing the development of the spatial organisation of non-linear responses across the fronto-temporal cortex. Around 5 months of age, bilateral inferior frontal channels showed responses consistent with sub-additive responses, while bilateral superior temporal channels showed different non-linear responses depending on the hemisphere: the left super-additive-consistent while the right sub-additive-consistent responses.

As we measured the fronto-temporal responses bilaterally, we were able to draw conclusions regarding the development of the lateralisation of speech integration, showing that integration is initially supported predominantly by the right hemisphere. Consistent with the majority univariate responses observed over the right hemisphere, when we used the network approach (MVPA), we found successful classification only in the right hemisphere. Analysis of relative channel weights showed that right inferior frontal and superior temporal channels were amongst the most informative to the successful classification (of responses to bimodal versus alternating unimodal conditions). Such result indicates that either integration is initially predominantly supported by the right fronto-temporal regions, or the right hemisphere shows a more stable/predictable pattern of responses than the left hemisphere around 5 months of age. This is consistent with previous EEG studies which found audiovisual mismatch responses, i.e., differential responses to congruent versus incongruent audiovisual syllables, over the right hemisphere at 5 months of age (Kushnerenko et al. 2008).

Surprisingly, we found that around 10 months of age the superior temporal but not the inferior frontal region showed non-linear responses to integration of congruent audiovisual speech. To the best of our knowledge, this was the first study to measure inferior frontal responses to audiovisual speech in infants above 8 months of age. As such we extended previous findings which showed inferior frontal sensitivity to integration at 5 months, showing no evidence of inferior frontal sensitivity to integration around 10 months of age. The observed superior temporal responses extend previous findings - the superior temporal region showing greater responses to incongruent than congruent audiovisual speech (Ujiie et al. 2020) - by showing the non-linear responses to integration of congruent audiovisual speech. Interestingly, in the older age group MVPAs - despite arguably providing greater sensitivity than univariate analyses (Emberson et al. 2017) - did not classify responses to bimodal (audiovisual) and alternating unimodal (auditory + visual) speech above the chance level. Unlike MVPA in fMRI, MVPA in infant fNIRS often relies on averaging across trials to establish a reliable estimate of neural patterns and subsequent across-participant decoding (Emberson et al. 2017). Nevertheless, it provides valuable and complementary information to the univariate analyses. Possibly, some infants showed a very specific pattern of responses while others did not. Lack of successful classification may reflect the fact that brain activation patterns are not reliable across participants and/or across trials at this age.

Given findings from the younger and older age groups - widespread, bilateral fronto-temporal responses in the younger age group and focal, mostly superior temporal responses in the older age group - it is possible that the period around 10 months of age involves a re-organisation of the cortical correlates of integration. Previous studies also show no or decreased distinct cortical responses to speech and social stimuli in infants around ten months of age, as compared to younger or older infants (e.g., Fava et al. 2014; Lloyd-Fox et al. 2017; Minagawa-Kawai et al. 2007). Fava et al. (2014) found differential left hemisphere responses to native than non-native audiovisual speech only at 11 to 14 months of age but not at 7 to 10 months of age. In a study on social motion, Lloyd-Fox et al. (2017) found similar response patterns in younger and older age groups (4–8 and 12–16 months of age), but fewer selective channels in 9- to 13-month-olds. In a study on discrimination of vowel-length changes, Minagawa-Kawai et al. (2007) found significantly different cortical responses to within- and across-category changes in 6- to 7- and 13- to 14-month-olds, but not 10- to 11-month-olds. While detailed longitudinal or cross-sectional work is lacking, findings from EEG studies suggest that audiovisual speech integration becomes left-lateralised in the second half of the first year of life: The audiovisual mismatch response to McGurk stimuli was observed mainly over the right hemisphere at 5 months (Kushnerenko et al. 2008) and over the left temporal region at 12 months of age (Riva et al. 2022). Together, these results indicate that the period around ten months of age is a transition period during which the fronto-temporal speech network (and likely other areas) becomes re-organised. This apparent period of re-organisation informs our understanding of the development of functional specialisation of the cortex, possibly reflecting a developmental pattern that occurs around 9 to 10 months of age. In that, between 9 and 10 months of age, cortical responses become less stable across infants, leading to less observed activations at the group level. Studies investigating structural brain development and functional connectivity are needed to further examine the hypothesis that 9–10 months of age is a period of re-organisation of the cortical networks for audiovisual speech processing.

### Limitations

The present study has several methodological strengths, including multiple age groups, a wide area of the cortex covered (bilateral fronto-temporal), a very strong experimental contrast (bimodal versus alternating unimodal condition), including a control for low-level processing within the baseline. Nevertheless, the current study needs to be viewed in light of some limitations. Most of the presented results did not survive the correction for multiple comparisons. Such correction is favourable in studies running channel-by-channel analyses, to counteract the effect of the high number of statistical tests being performed, which increases the possibility of false-positives. Another approach to deal with false positives is to check for adjacent channels. The statistical likelihood of two or more adjacent channels producing false-positive results is very low (p = .013) (Lloyd-Fox et al. 2011), and in our study most channels that showed a significant effect were adjacent. Therefore it is unlikely that the presented results reflect false positives. We argue that the presented results are informative, even if they should be interpreted with caution and replicated in future work.

We draw conclusions regarding the pattern of cortical responses consistent with super- versus sub-additive responses based on the observed patterns of differential responses to the bimodal (audiovisual) versus alternating unimodal (auditory + visual) conditions. While this approach has been previously used in adult studies (Olson et al. 2002), it does not follow the original experimental approach proposed by Stein and colleagues (1983). However, neuroimaging studies with infants have to negotiate between attrition and experimental design. By limiting the number of conditions we were able to decrease the testing time, thus increasing the number of infants that contributed enough data (Hoehl & Wahl, 2012). Furthermore, multisensory stimuli are more attention-grabbing (e.g., Reynolds and Guy 2012), thus by having both conditions include both auditory and visual stimuli, we likely increased the amount of data infants contributed. Having an auditory-only and visual-only conditions may be problematic as infants might attend to these blocks less relative to the audiovisual blocks (a big problem for fNIRS studies that require infants to attend to the stimuli for at least 4-6s). To disentangle the meaning of the observed sub-additive-consistent responses, future infant studies could measure cortical responses to audiovisual, visual, and auditory speech separately (as done in adult studies, e.g., Venezia et al. 2017).

The conclusions regarding the age-related changes in cortical specialisation could reflect noisy data rather than a meaningful developmental change. We observed fewer active channels in the older than younger age group, and significant classification accuracy only in the younger age group. Direct comparison between age groups showed no significant effect of age on channels showing significant responses in either age group (see Supplementary Materials). Both the univariate and MVPA results could reflect increased variability in observed responses in the older age group, leading to lower power to detect significant effects. This variability may be related to the COVID-19 pandemic, which started during data collection. A third of the infants in the older age group were tested while COVID-related restrictions were in place. Exposure to people wearing masks could have impacted infants’ processing of audiovisual speech, as visual speech cues were less accessible. In adults, a month of mask-wearing (enforced by the country COVID-19-related procedures) influenced perception of a well-known audiovisual speech illusion, the McGurk illusion (Chládková et al. 2021). If a month can change an otherwise stable perception in adults, a few months likely changed the learning trajectory of how infants process talking faces. On the other hand, infants may have gained more exposure to talking faces at home, as families are working from home and schools are closed. Future studies should look at the effects of the pandemic on the development of infants’ knowledge about audiovisual speech.

## Conclusions

The current study extends previous findings on the neural correlates of audiovisual integration by showing the development of non-linear responses to integration of congruent audiovisual speech. Instead of a gradual emergence of non-linear responses to integration of speech, our results show that the development of integration of audiovisual speech is a complex process. Already around 5 months of age we observed widespread non-linear responses to integration in bilateral fronto-temporal regions. This result is consistent with adult studies (e.g., Calvert et al. 2000) showing non-linear responses to speech integration, indicating an early cortical specialization. While in adults the responses to integration of congruent speech are typically super-additive (e.g., Calvert et al. 2000), in infants channel-by-channel analyses revealed responses consistent with both super- and sub-additive responses. The patterns of fronto-temporal responses were decodable around 5 months of age, indicating that audiovisual speech elicits different patterns of brain responses than alternating auditory and visual speech. Consistent with the channel-wise analyses, the analysis of relative channel weights fro MVPA showed that the successful decoding was driven by the right hemisphere. Specifically, channels in the right inferior frontal and superior temporal regions showed non-linear responses and were most informative for MVPA, indicating that around 5 months of age integration is predominantly supported by the right hemisphere. Interestingly, despite the increasing behavioral and cortical specialization for processing native speech (e.g., Werker and Tees 1984; Fava et al. 2014), the predominance of responses consistent with a super-additive response was not observed even by 10 months of age. Around 10 months of age the network becomes increasingly focal, consistently with previous work showing that increasing cortical specialization is related to smaller areas showing differential responses (e.g., Lloyd-Fox et al. 2017). However, at the same time the distributed patterns of activation – decodable around 5 months of age – become undecodable for bimodal (audiovisual) congruent versus alternating unimodal (alternating audio and visual) articulation. This may reflect either the re-organisation of the network supporting integration resulting either from less stable/predictable patterns of responses within infants or high variability in the patterns of responses between infants. As such, this study further contributes to the growing body of research on the functional development of the cortex in infancy, by showing the developmental changes in HbO and HbR responses to integration of audiovisual speech over the fronto-temporal regions. Future studies with atypically developing infants will benefit from an increased understanding of the development of functional specialisation for multisensory speech in infancy.

## Electronic Supplementary Material

Below is the link to the electronic supplementary material.

Supplementary Material 1

## Footnotes

1 15% of infants in the younger age group and 32% of infants in the older age group were tested during the COVID-19 pandemic.

## References

[CR1] Aldridge MA, Braga ES, Walton GE, Bower TGR (1999). The intermodal representation of speech in newborns. Dev Sci.

[CR3] Altvater-Mackensen N, Grossmann T (2016). The role of left inferior frontal cortex during audiovisual speech perception in infants. NeuroImage.

[CR5] Altvater-Mackensen N, Grossmann T (2018). Modality-independent recruitment of inferior frontal cortex during speech processing in human infants. Dev Cogn Neurosci.

[CR75] Ben, Deen Hilary, Richardson Daniel D., Dilks Atsushi, Takahashi Boris, Keil Lawrence L., Wald Nancy, Kanwisher Rebecca, Saxe (2017) Organization of high-level visual cortex in human infants Abstract Nature Communications 8(1) 10.1038/ncomms13995 10.1038/ncomms13995 PMC5234071 28072399

[CR7] Benjamini Y, Hochberg Y (1995) Controlling the false Discovery rate: a practical and powerful Approach to multiple testing. J Roy Stat Soc: Ser B (Methodol) 57(1). 10.1111/j.2517-6161.1995.tb02031.x

[CR8] Brainard DH (1997) The Psychophysics Toolbox.Spatial Vision, *10*(4) 9176952

[CR10] Callan DE, Jones JA, Munhall KG, Callan AM, Kroos C, Vatikiotis-Bateson E (2003). Neural processes underlying perceptual enhancement by visual speech gestures. NeuroReport.

[CR11] Calvert GA, Campbell R, Brammer MJ (2000). Evidence from functional magnetic resonance imaging of crossmodal binding in the human heteromodal cortex. Curr Biol.

[CR12] Carnevali L, Gui A, Jones EJ, Farroni T (2022) Face processing in early development: a systematic review of behavioral studies and considerations in times of COVID-19 pandemic.Frontiers in Psychology,388 10.3389/fpsyg.2022.778247 PMC8894249 35250718

[CR13] Chládková K, Podlipský VJ, Nudga N, Šimáčková Å (2021). The McGurk effect in the time of pandemic: age-dependent adaptation to an environmental loss of visual speech cues. Psychonomic Bull Rev.

[CR14] Cristia A, Minagawa-Kawai Y, Egorova N, Gervain J, Filippin L, Cabrol D, Dupoux E (2014). Neural correlates of infant accent discrimination: an fNIRS study. Dev Sci.

[CR76] De Meo, R., Murray, M. M., Clarke, S., & Matusz, P. J. (2015). Top-down control and early multisensory processes: chicken vs. egg. Frontiers in integrative neuroscience, 9, 17. Chicago 10.3389/fnint.2015.00017 PMC4347447 25784863

[CR17] Dopierała AAW, Mercure E, Pluta A, López Pérez D, Wolak T, Tomalski P (2020), January 24 Development of neural correlates of audiovisual speech integration in infancy. An fNIRS study pre-registration. Retrieved from

[CR19] Duncan A, Meek JH, Clemence M, Elwell CE, Fallon P, Tyszczuk L, Cope M, Delpy DT (1996). Measurement of cranial optical path length as a function of age using phase resolved near infrared spectroscopy. Pediatr Res.

[CR20] Egorova N, Cristià A, Vendelin I, Filippin L, Long B, Gervain J, Minagawa-Kawai Y, Dupoux E (2010). Neural correlates of dialect perception in early infancy. J Acoust Soc Am.

[CR21] Emberson LL, Zinszer BD, Raizada RDSS, Aslin RN (2017). Decoding the infant mind: multivariate pattern analysis (MVPA) using fNIRS. PLoS ONE.

[CR22] Erickson LC, Zielinski BA, Zielinski JEV, Liu G, Turkeltaub PE, Leaver AM, Rauschecker JP (2014). Distinct cortical locations for integration of audiovisual speech and the McGurk effect. Front Psychol.

[CR23] Faul F, Erdfelder E, Lang A-G, Buchner A (2009) Statistical power analyses using G*Power 3.1: tests for correlation and regression analyses.Behavior Research Methods, *41*(4) 10.3758/BRM.41.4.1149 19897823

[CR24] Fava E, Hull R, Bortfeld H (2014). Dissociating cortical activity during processing of native and non-native audiovisual speech from early to late infancy. Brain Sci.

[CR25] Gervain J, Macagno F, Cogoi S, Pena M, Mehler J (2008) The neonate brain detects speech structure. *Proceedings of the National Academy of Sciences*, *105*(37), 14222–14227. doi:10.1073/pnas.0806530105 10.1073/pnas.0806530105 PMC2544605 18768785

[CR26] Grossmann T, Oberecker R, Koch SP, Friederici AD (2010). The Developmental Origins of Voice Processing in the human brain. Neuron.

[CR27] Haxby JV, Gobbini MI (2012) Distributed Neural Systems for Face Perception. *Oxford Handbook of Face Perception*, 93–110. doi:10.1093/oxfordhb/9780199559053.013.0006

[CR28] Haxby JV, Gobbini MI, Furey ML, Ishai A, Schouten JL, Pietrini P (2001) Distributed and overlapping representations of faces and objects in ventral temporal cortex. Science 293(5539). 10.1126/science.1063736 10.1126/science.1063736 11577229

[CR29] Haynes JD, Rees G (2006). Decoding mental states from brain activity in humans. Nat Rev Neurosci.

[CR77] Hoehl, S., & Wahl, S. (2012). Recording infant ERP data for cognitive research. Developmental neuropsychology, 37(3), 187–209. 10.1080/87565641.2011.627958 22545658

[CR30] Homae F, Watanabe H, Nakano T, Taga G (2007). Prosodic processing in the developing brain. Neurosci Res.

[CR18] https:// osf.io/exhdb/?view_only=df6e53398cbb46f0a7437d7d04d89fed

[CR31] Huang P, Zhou F, Guo Y, Yuan S, Lin S, Lu J, Tu S, Lu M, Shen S, Guedeney A, Xia H (2021) Association between the COVID-19 pandemic and infant neurodevelopment: a comparison before and during COVID-19.Frontiers in pediatrics,1087 10.3389/fped.2021.662165 PMC8527007 34692602

[CR32] Huppert TJ, Diamond SG, Franceschini MA, Boas DA (2009). HomER: a review of time-series analysis methods for near-infrared spectroscopy of the brain. Appl Opt.

[CR34] Hyde DC, Flom R, Porter CL (2016). Behavioral and neural foundations of multisensory face-voice perception in infancy. Dev Neuropsychol.

[CR33] Hyde DC, Jones BL, Flom R, Porter CL (2011). Neural signatures of face–voice synchrony in 5-month‐old human infants. Dev Psychobiol.

[CR35] Hyde DC, Jones BL, Porter CL, Flom R (2010). Visual stimulation enhances auditory processing in 3-month‐old infants and adults. Dev Psychobiology: J Int Soc Dev Psychobiol.

[CR36] Kleiner M, Brainard DH, Pelli DG, Broussard C, Wolf T, Niehorster D (2007) What’s new in Psychtoolbox-3? *Perception*, *36*

[CR37] Kopp F (2014). Audiovisual temporal fusion in 6-month-old infants. Dev Cogn Neurosci.

[CR38] Kopp F, Dietrich C (2013). Neural dynamics of audiovisual synchrony and asynchrony perception in 6-month-old infants. Front Psychol.

[CR40] Kushnerenko E, Tomalski P, Ballieux H, Ribeiro H, Potton A, Axelsson EL, Murphy E, Moore DG (2013). Brain responses to audiovisual speech mismatch in infants are associated with individual differences in looking behaviour. Eur J Neurosci.

[CR39] Kushnerenko EV, Teinonen T, Volein A, Csibra G (2008). Electrophysiological evidence of illusory audiovisual speech percept in human infants. Proc Natl Acad Sci USA.

[CR42] Lewkowicz DJ (2010). Infant perception of audio-visual speech synchrony. Dev Psychol.

[CR43] Lewkowicz DJ, Ghazanfar AA (2009), November The emergence of multisensory systems through perceptual narrowing. *Trends in Cognitive Sciences*, Vol 13, bll 470–478. doi:10.1016/j.tics.2009.08.004 10.1016/j.tics.2009.08.004 19748305

[CR44] Lloyd-Fox S, Begus K, Halliday D, Pirazzoli L, Blasi A, Papademetriou M, Darboe MK, Prentice AM, Johnson MH, Moore SE, Elwell CE (2017). Cortical specialisation to social stimuli from the first days to the second year of life: a rural gambian cohort. Dev Cogn Neurosci.

[CR47] Lloyd-Fox S, Blasi A, Elwell CE (2010). Illuminating the developing brain: the past, present and future of functional near infrared spectroscopy. Neurosci Biobehav Rev.

[CR48] Lloyd-Fox S, Blasi A, Everdell N, Elwell CE, Johnson MH (2011). Selective cortical mapping of biological motion processing in young infants. J Cogn Neurosci.

[CR46] Lloyd-Fox S, Blasi A, Volein A, Everdell N, Elwell CE, Johnson MH (2009). Social perception in infancy: a near infrared spectroscopy study. Child Dev.

[CR49] Lloyd-Fox S, Papademetriou M, Darboe MK, Everdell NL, Wegmuller R, Prentice AM, Moore SE, Elwell CE (2014). Functional near infrared spectroscopy (fNIRS) to assess cognitive function in infants in rural Africa. Sci Rep.

[CR45] Lloyd-Fox S, Széplaki-Köllőd B, Yin J, Csibra G (2015). Are you talking to me? Neural activations in 6-month-old infants in response to being addressed during natural interactions. Cortex.

[CR50] Matchin W, Groulx K, Hickok G (2014). Audiovisual Speech Integration does not rely on the Motor System: evidence from Articulatory suppression, the McGurk Effect, and fMRI. J Cogn Neurosci.

[CR51] Mercure E, Evans S, Pirazzoli L, Goldberg L, Bowden-Howl H, Coulson-Thaker K, Beedie I, Lloyd-Fox S, Johnson MH, MacSweeney M (2020). Language experience impacts brain activation for spoken and signed language in infancy: insights from unimodal and bimodal bilinguals. Neurobiol Lang.

[CR52] Meredith M, Stein B (1983). Interactions among converging sensory inputs in the superior colliculus. Science.

[CR53] Minagawa-Kawai Y, Mori K, Naoi N, Kojima S (2007). Neural attunement processes in infants during the Acquisition of a Language-Specific Phonemic contrast. J Neurosci.

[CR54] Molavi B, Dumont GA (2012). Wavelet-based motion artifact removal for functional near-infrared spectroscopy. Physiol Meas.

[CR55] Naoi N, Minagawa-Kawai Y, Kobayashi A, Takeuchi K, Nakamura K, Yamamoto JI, Kojima S (2012). Cerebral responses to infant-directed speech and the effect of talker familiarity. NeuroImage.

[CR57] Olson IR, Gatenby JC, Gore JC (2002) *A comparison of bound and unbound audio-visual information processing in the human cerebral cortex* (Vol 14). Presented at the Cognitive Brain Research. doi:10.1016/S0926-6410(02)00067-8 10.1016/s0926-6410(02)00067-8 12063136

[CR58] Patterson ML, Werker JF (2002). Infants’ ability to match dynamic phonetic and gender information in the face and voice. J Exp Child Psychol.

[CR59] Pelli DG (1997) The VideoToolbox software for visual psychophysics: transforming numbers into movies. Spat Vis 10(4). 10.1163/156856897X00366 9176953

[CR60] Pereira F, Mitchell T, Botvinick M (2009). Machine learning classifiers and fMRI: a tutorial overview. NeuroImage.

[CR63] Reynolds GD, Guy MW (2012). Brain–behavior relations in infancy: integrative approaches to examining infant looking behavior and event-related potentials. Dev Neuropsychol.

[CR62] Reynolds LC, Pineda RG, Mathur A, Vavasseur C, Shah DK, Liao S, Inder T (2014). Cerebral maturation on amplitude-integrated electroencephalography and perinatal exposures in preterm infants. Acta Paediatr.

[CR64] Riva V, Riboldi EM, Dondena C, Piazza C, Molteni M, Cantiani C (2022). Atypical ERP responses to audiovisual speech integration and sensory responsiveness in infants at risk for autism spectrum disorder. Infancy.

[CR16] Di Lorenzo R, Pirazzoli L, Blasi A, Bulgarelli C, Hakuno Y, Minagawa Y, Brigadoi S (2019) Recommendations for motion correction of infant fNIRS data applicable to multiple data sets and acquisition systems NeuroImage 200(June):511–527. 10.1016/j.neuroimage.2019.06.056. 10.1016/j.neuroimage.2019.06.056 31247300

[CR65] Rosenblum LD, Schmuckler MA, Johnson JA (1997) The McGurk effect in infants. *Perception & psychophysics*, *59*(3), 347–357. Retrieved from http://eutils.ncbi.nlm.nih.gov/entrez/eutils/elink.fcgi?dbfrom=pubmed&id=9136265&retmode=ref&cmd=prlinks 10.3758/bf03211902 9136265

[CR66] Ruxton GD, Neuhäuser M (2013). Review of alternative approaches to calculation of a confidence interval for the odds ratio of a 2 × 2 contingency table. Methods Ecol Evol.

[CR67] Scholkmann F, Spichtig S, Muehlemann T, Wolf M (2010). How to detect and reduce movement artifacts in near-infrared imaging using moving standard deviation and spline interpolation. Physiol Meas.

[CR68] Stein BE, Meredith MA (1993) *Cognitive neuroscience. The merging of the senses*

[CR69] Ujiie Y, Kanazawa S, Yamaguchi MK (2020). The other-race-effect on Audiovisual Speech Integration in Infants: a NIRS Study. Front Psychol.

[CR71] Venezia JH, Vaden KI, Rong F, Maddox D, Saberi K, Hickok G (2017). Auditory, visual and Audiovisual Speech Processing Streams in Superior temporal sulcus. Front Hum Neurosci.

[CR72] Werchan DM, Baumgartner HA, Lewkowicz DJ, Amso D (2018). The origins of cortical multisensory dynamics: evidence from human infants. Dev Cogn Neurosci.

[CR73] Werker JF, Tees RC (1984). Cross-language speech perception: evidence for perceptual reorganization during the first year of life. Infant Behav Dev.

[CR74] Wright TM, Pelphrey KA, Allison T, McKeown MJ, McCarthy G (2003). Polysensory interactions along lateral temporal regions evoked by audiovisual speech. Cereb Cortex.

