# Supplementary material for: The Development of Cortical Responses to the Integration of Audiovisual Speech in Infancy
Source: Brain Topogr. 2023 May 12;36(4):459–75. doi: 10.1007/s10548-023-00959-8 (PMC10176292; doi:10.1007/s10548-023-00959-8)
Supplement: Supplementary file 1 — Supplementary Material 1 [file 10548_2023_959_MOESM1_ESM.docx]

**Supplementary Material**

*Table S1.* Classification accuracy and significance level for MVPAs of all, left and right hemisphere channels in younger (N=20) and older (N=21) age groups.

|  | | All channels | |  | Left hemisphere | |  | Right hemisphere | |
| --- | --- | --- | --- | --- | --- | --- | --- | --- | --- |
| Chrom | TW | proportion correct | *p* |  | proportion correct | *p* |  | proportion correct | *p* |
| Younger age group | | |  |  |  |  |  |  |  |
| HbO | 5–10s | .43 | *> .8* |  | .43 | *> .8* |  | .48 | *> .7* |
|  | 10–15s | .63 | *> .1* |  | .55 | *> .4* |  | .68* | *.044* |
| HbR | 5–10s | .55 | *> .3* |  | .45 | *> .7* |  | .53 | *> .4* |
|  | 10–15s | .63 | *> .1* |  | .5 | *> .5* |  | .65 | *> .07* |
| Older age group | | | | | | | | | |
| HbO | 5–10s | .43 | *> .8* |  | .41 | *> .8* |  | .46 | *> .7* |
|  | 10–15s | .48 | *> .6* |  | .46 | *> .7* |  | .5 | *> .5* |
| HbR | 5–10s | .46 | *> .7* |  | .55 | *> .3* |  | .46 | *> .7* |
|  | 10–15s | .43 | *> .8* |  | .52 | *> .4* |  | .36 | *> .9* |

*Note*. Chrom, chromophore, * p < .05.

*Table S2. Exploratory MVPAs without infants tested during the COVID-19 pandemic.* Classification accuracy and significance level for MVPAs of all, left and right hemisphere channels in younger (N=17) and older (N=15) age groups.

|  | | All channels | |  | Left hemisphere | |  | Right hemisphere | |
| --- | --- | --- | --- | --- | --- | --- | --- | --- | --- |
| Chrom | TW | proportion correct | *p* |  | proportion correct | *p* |  | proportion correct | *p* |
| Younger age group | | |  |  |  |  |  |  |  |
| HbO | 5–10s | .38 | > .9 |  | .44 | > .7 |  | .41 | > .9 |
|  | 10–15s | .56 | > .3 |  | .5 | > .5 |  | .71* | .034 |
| HbR | 5–10s | .47 | > .66 |  | .56 | > .3 |  | .5 | > .5 |
|  | 10–15s | .59 | > .2 |  | .56 | > .3 |  | .62 | > .1 |
| Older age group | | | | | | | | | |
| HbO | 5–10s | .47 | > .6 |  | .6 | > .1 |  | .47 | > .7 |
|  | 10–15s | .57 | > .3 |  | .63 | > .1 |  | .5 | > .5 |
| HbR | 5–10s | .57 | > .3 |  | .63 | > .09 |  | .4 | > .8 |
|  | 10–15s | .53 | > .4 |  | .53 | > .4 |  | .43 | > .7 |

*Note*. Chrom, chromophore, * p < .05.

# Sensitivity to synchronous audiovisual speech and asynchronous auditory/visual speech

We used planned simple contrasts to analyse which channels are active to each condition (synchronous audiovisual speech, asynchronous auditory/visual speech) relative to baseline (see Table S3). We report channels showing both typical (increase in HbO and/or decrease in HbR) and atypical (decrease in HbO and/or increase in HbR) responses.

*Table S3.* Channels showing a significant response to the asynchronous auditory/visual and synchronous audiovisual speech conditions (relative to baseline) in both age groups. Planned simple contrasts.

| Chrom | H | ROI | CH | TW | *F* | *df* | *df_(Error)_* | *p* | 𝜂*^2^_p_* | ↑↓ |
| --- | --- | --- | --- | --- | --- | --- | --- | --- | --- | --- |
| Younger age group | | | |  |  |  |  |  |  |  |
| Asynchronous auditory/visual speech | | | | | | |  |  |  |  |
| HbO | R | IF | 24* | 10-15s | 15.907 | 1 | 19 | <.001 | .456 | ↑ |
|  | R | IF | 26* | 10-15s | 16.757 | 1 | 17 | <.001 | .496 | ↑ |
|  | R | ST | 29 | 10-15s | 6.219 | 1 | 16 | .024 | .28 | ↑ |
|  | R | ST | 33 | 10-15s | 9.052 | 1 | 17 | .008 | .347 | ↑ |
|  | R | ST | 34 | 10-15s | 5.627 | 1 | 18 | .029 | .238 | ↑ |
|  | R | ST | 38 | 10-15s | 5.204 | 1 | 19 | .034 | .215 | ↑ |
|  | R | - | 42 | 10-15s | 4.867 | 1 | 19 | .04 | .204 | ↑ |
| HbR | L | ST | 7 | 10-15s | 5.8 | 1 | 13 | .032 | .309 | ↓ |
|  | R | IF | 24 | 5-10s | 6.657 | 1 | 19 | .018 | .259 | ↑ |
|  | R | IF | 26 | 5-10s | 4.782 | 1 | 17 | .043 | .22 | ↑ |
|  | R | IF | 27 | 5-10s | 4.526 | 1 | 17 | .048 | .21 | ↑ |
|  | R | ST | 29 | 5-10s | 6.277 | 1 | 16 | .023 | .282 | ↑ |
|  | R | - | 44 | 10-15s | 5.076 | 1 | 19 | .036 | .211 | ↑ |
|  | R | - | 45 | 5-10s | 8.949 | 1 | 19 | .008 | .32 | ↑ |
| Synchronous audiovisual speech | | | | | |  |  |  |  |  |
| HbO | L | ST | 10 | 10-15s | 6.089 | 1 | 19 | .023 | .243 | ↑ |
|  | L | ST | 11 | 10-15s | 7.38 | 1 | 17 | .015 | .303 | ↑ |
| HbR | L | IF | 3 | 10-15s | 4.408 | 1 | 18 | .05 | .197 | ↑ |
|  | L | IF | 4 | 5-10s | 5.304 | 1 | 18 | .033 | .228 | ↑ |
|  | L | ST | 10 | 5-10s | 5.706 | 1 | 19 | .027 | .231 | ↑ |
|  | L | ST | 12 | 5-10s | 6.325 | 1 | 17 | .022 | .271 | ↑ |
|  | L | - | 20 | 5-10s | 5.995 | 1 | 17 | .025 | .261 | ↑ |
|  | R | - | 43 | 10-15s | 4.831 | 1 | 19 | .041 | .203 | ↑ |
|  | R | - | 45 | 5-10s | 5.508 | 1 | 19 | .03 | .225 | ↑ |
|  | R | - | 45 | 10-15s | 4.552 | 1 | 19 | .046 | .193 | ↑ |
| Older age group | | | |  |  |  |  |  |  |  |
| Asynchronous auditory/visual speech | | | | | | |  |  |  |  |
| HbO | L | IF | 1 | 10-15s | 6.89 | 1 | 21 | .016 | .247 | ↑ |
|  | L | IF | 3 | 10-15s | 8.484 | 1 | 21 | .008 | .288 | ↑ |
|  | R | IF | 26 | 10-15s | 5.62 | 1 | 20 | .028 | .219 | ↑ |
|  | R | - | 41 | 10-15s | 11.452 | 1 | 19 | .003 | .376 | ↑ |
|  | R | - | 42 | 10-15s | 4.579 | 1 | 21 | .044 | .179 | ↑ |
| HbR | L | ST | 15 | 10-15s | 7.29 | 1 | 18 | .015 | .288 | ↓ |
|  | L | - | 23 | 10-15s | 5.191 | 1 | 19 | .034 | .215 | ↑ |
|  | R | IF | 25 | 5-10s | 5.458 | 1 | 20 | .03 | .214 | ↑ |
|  | R | ST | 30 | 10-15s | 6.09 | 1 | 19 | .023 | .243 | ↓ |
|  | R | ST | 35 | 10-15s | 4.702 | 1 | 19 | .043 | .198 | ↑ |
|  | R | - | 44 | 5-10s | 4.73 | 1 | 20 | .042 | .191 | ↑ |
| Synchronous audiovisual speech | | | | | |  |  |  |  |  |
| HbO | L | IF | 4 | 10-15s | 7.496 | 1 | 20 | .013 | .273 | ↑ |
|  | L | ST | 10 | 10-15s | 9.646 | 1 | 21 | .005 | .315 | ↑ |
|  | R | ST | 33 | 10-15s | 6.646 | 1 | 18 | .019 | .27 | ↑ |
|  | R | ST | 34 | 10-15s | 7.554 | 1 | 20 | .012 | .274 | ↑ |
|  | R | - | 41 | 10-15s | 6.906 | 1 | 19 | .017 | .267 | ↑ |
| HbR | L | IF | 1 | 5-10s | 8.858 | 1 | 21 | .007 | .297 | ↑ |
|  | L | IF | 3 | 5-10s | 5.503 | 1 | 21 | .029 | .208 | ↑ |
|  | L | IF | 4 | 5-10s | 7.396 | 1 | 20 | .013 | .27 | ↑ |
|  | R | IF | 24 | 5-10s | 4.573 | 1 | 21 | .044 | .179 | ↑ |
|  | R | ST | 29 | 5-10s | 5.677 | 1 | 18 | .028 | .24 | ↑ |
|  | R | ST | 33 | 5-10s | 5.308 | 1 | 18 | .033 | .228 | ↑ |

*Note.* Chrom, chromophore, H, hemisphere, TW, time window. Arrows indicate direction of mean HbO/HbR concentration change between baseline and activation time window. * result significant after the FDR correction for multiple comparisons.

# Age-related differences in sensitivity to synchronous audiovisual speech and asynchronous auditory/visual speech

We used planned simple contrasts to analyse which channels show different responses to each condition (synchronous audiovisual speech, asynchronous auditory/visual speech) depending on age (5-6.5 months, 9-10.5 months) (see Table S4).

*Table S4.* Effect of age on responses to synchronous audiovisual speech and asynchronous auditory/visual speech. Planned simple contrasts.

| Chrom | H | ROI | CH | TW | F | df | df_(Error)_ | p | 𝜂^2^_p_ |
| --- | --- | --- | --- | --- | --- | --- | --- | --- | --- |
| Asynchronous auditory/visual speech | | | | | | | | | |
| HbO | L | ST | 12 | 5–10s | 5.352 | 1 | 39 | .026 | .121 |
|  | R | ST | 27 | 5–10s | 4.987 | 1 | 38 | .032 | .116 |
|  |  |  |  | 10–15s | 8.581 | 1 | 38 | .006 | .184 |
|  | R | ST | 33* | 10–15s | 4.91 | 1 | 35 | .033 | .123 |
| HbR | L | - | 20* | 5–10s | 4.787 | 1 | 37 | .035 | .115 |
|  | R | ST | 27* | 5–10s | 5.896 | 1 | 38 | .02 | .134 |
|  | R |  |  | 10–15s | 5.315 | 1 | 38 | .027 | .123 |
|  | R | ST | 38* | 5–10s | 4.565 | 1 | 39 | .039 | .105 |
| Synchronous audiovisual speech | | | | | | | | | |
| HbO | L | ST | 10* | 5–10s | 5.425 | 1 | 40 | .025 | .119 |
|  | L | ST | 11* | 5–10s | 5.409 | 1 | 38 | .025 | .125 |
|  | R | IF | 24* | 10–15s | 4.868 | 1 | 40 | .033 | .108 |
| HbR | L | ST | 11* | 5–10s | 4.797 | 1 | 38 | .035 | .112 |
|  | R | IF | 24* | 10–15s | 5.155 | 1 | 40 | .029 | .114 |
|  | R | - | 43* | 10–15s | 5.07 | 1 | 40 | .03 | .112 |

*Note.* Chrom, chromophore, H, hemisphere, CH, channel number, TW, time window. No results survived the FDR correction for multiple comparisons.
